# Mobilization of hematopoietic stem cells with lenograstim in multiple myeloma patients: Prospective multicenter observational study (KMM122)

**DOI:** 10.1002/cam4.5657

**Published:** 2023-03-23

**Authors:** Eun Hee Jung, Ja Min Byun, Dong‐Yeop Shin, Young Rok Do, Jae‐Cheol Jo, Sang Min Lee, Sung‐Soo Yoon

**Affiliations:** ^1^ Department of Internal Medicine Seoul National University Bundang Hospital Seongnam South Korea; ^2^ Department of Internal Medicine Seoul National University Hospital Seoul South Korea; ^3^ Cancer Research Institute Seoul National University College of Medicine Seoul South Korea; ^4^ Center for Medical Innovation, Biomedical Research Institute Seoul National University Hospital Seoul South Korea; ^5^ Department of Internal Medicine Dongsan Medical Center, Keimyung University School of Medicine Daegu South Korea; ^6^ Department of Hematology and Oncology Ulsan University Hospital, University of Ulsan College of Medicine Ulsan South Korea; ^7^ Department of Internal Medicine, Busan Paik Hospital Inje University College of Medicine Busan South Korea

**Keywords:** autologous hematopoietic stem cell transplantation, G‐CSF, lenograstim, mobilization, multiple myeloma

## Abstract

**Background:**

Current guidelines recommend using filgrastim or tbo‐filgrastim to mobilize hematopoietic progenitor cells in an autologous setting. However, previous studies have suggested other forms of granulocyte colony‐stimulating factor (G‐CSF) are equally efficacious, possibly with fewer leukaphereses required. Thus, we prospectively studied the efficacy of lenograstim, a glycosylated recombinant form of G‐CSF, in multiple myeloma (MM) patients.

**Methods:**

From November 2011 to January 2020, 98 MM patients undergoing autologous stem cell transplant (ASCT) from five academic centers in Korea were enrolled. Patients were mobilized with subcutaneous lenograstim (Neutrogin®) with fixed doses of 10 μg/kg for 4 days.

**Results:**

Most of the patients ( *N*  = 90, 91.8%) achieved at least the targets of 2 × 10^6^ CD34+ cells/kg body weight, and more than half of MM patients ( *N*  = 57, 58.2%) reached a target of 5 × 10^6^ CD34+ cells/kg body weight. The mobilization failure rate was 8.2% ( *N*  = 8). The median number of CD34 + cell/kg using G‐CSF only was 5.25 × 10^6^/kg (range 0.49–13.47). Adverse events included transfusion (TF, *N*  = 53, 54.1%), bone pain ( *N*  = 6, 6.1%), fever ( *N*  = 2, 2.0%), and gastrointestinal troubles ( *N*  = 2, 2.0%). There were no grade 3 or 4 adverse events during mobilization. Body surface area (BSA) at mobilization and platelet TF were factors associated with CD34+ collection. Most patients achieved neutrophil ( *N*  = 93, 98.9%) and platelet ( *N*  = 89, 95.7%) engraftment.

**Conclusion:**

Lenograstim can safely and effectively mobilize stem cells in MM autologous settings.

## Graphical abstract

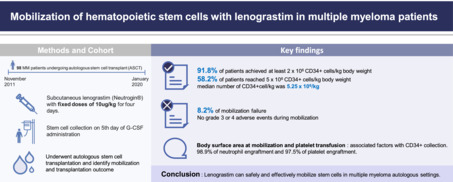

## 1 INTRODUCTION

Autologous stem cell transplantation (ASCT) remains an integral part of multiple myeloma (MM) treatment even in the midst of the evolution of the therapeutic arsenal for MM.^1^ As adequate peripheral blood stem cell (PBSC) collection is crucial for successful ASCT, wise selection of the mobilization regimen has garnered a lot of interest over the years.^2^, ^3^

Currently, granulocyte colony‐stimulating factor (G‐CSF) is used alone or following chemotherapy for PBSC mobilization in MM. G‐CSF induces the proliferation and differentiation of myeloid precursor cells and influences the chemotaxis and antigen expression of neutrophils. There are three G‐CSF preparations available for mobilization. First, lenograstim (glycosylated rHu G‐CSF) is obtained from Chinese hamster ovarian cells, and consists of 174 amino acids with 4% glycosylation.^4^ Secondly, filgrastim (nonglycosylated Hu G‐CSF) is produced using *Escherichia coli* and has a methionine group at its N‐terminal end.^5^ Lastly, pegfilgrastim (pegylated form of nonglycosylated Hu G‐CSF) is obtained by the attachment of the polyethylene glycol (PEG) moiety.^4^

Interestingly, the latest NCCN guideline^6^ only mentions filgrastim and pegfilgrastim as the preferred G‐CSF preparations for mobilization. Although several retrospective studies are comparing the PBSC mobilizing efficacy of glycosylated versus non‐glycosylated G‐CSF,^6^, ^7^, ^8^, ^9^, ^10^ they have failed to reach a unanimous conclusion. To this end, we aimed to prospectively examine the efficacy and safety of lenograstim‐only mobilization in a homogeneous population.

## 2 PATIENTS AND METHODS

### 2.1 Study overview

This was a multicenter, prospective observational study. The primary objective of the study was to examine the optimal mobilization rate, defined as a collection of ≥5 × 10^6^ CD34+ cells/kg body weight within four rounds of harvest apheresis. Secondary objectives included (1) adequate mobilization rate, defined as collection of ≥2 × 10^6^ CD34+ cells/kg body weight (2) mobilization failure rate, defined as collection of <2 × 10^6^ CD34+ cells/kg body weight; (3) variables affecting mobilization; (4) safety of lenograstim mobilization; and (5) subsequent ASCT outcomes. Cutoffs of 2 × 10^6^ CD34+ cells/kg body weight and 5 × 10^6^ CD34+ cells/kg body weight were used because it is generally accepted that cell dose ≥2 × 10^6^ ensures a threshold for neutrophil engraftment during hematopoietic stem cell transplantation, while ≥5 × 10^6^ ensures a threshold for platelet engraftment.^11^

### 2.2 Study population

Symptomatic multiple myeloma patients aged between 20 and 65 years who achieved at least partial response per International Myeloma Working Group (IMWG)^12^ criteria were enrolled. Patients with a history of prior PBSC mobilization were excluded. Patients with ECOG>2 were also excluded. This study was conducted according to the Declaration of Helsinki and was approved by the Institutional Review Board of each participating hospital (Seoul National University Hospital, Ulsan University Hospital, Busan Paik Hospital, Keimyung University Dongsan Medical Center, and Korea Cancer Center Hospital). Informed consent was taken from all patients before participating in any study‐related procedure.

### 2.3 Mobilization and PBSC harvest

Lenograstim (Neutrogin®; JW Pharmaceutical Corporation, Seoul, Korea) was given at a fixed dose of 10 μg/kg for four consecutive days (Figure S1). No dose adjustment was allowed. PBSC harvest apheresis was carried out on Day 5 and onward. If CD34+ cells ≥2 × 10^6^/kg body weight were secured, the patient subsequently underwent ASCT after conditioning.

### 2.4 Statistical analysis

The Student's *t*‐test or Wilcoxon's sign rank test was used for continuous variables. Pearson's chi‐squared test or Fisher's exact were used for categorical variables. To identify variables that might affect mobilization, we used multiple linear regression models: with the stepwise backward procedure, predictors achieving a *P‐*value below 0.05 were considered then sequentially removed if the *P‐*value in the multiple model was above 0.05.

The study cutoff was Sept 1 2020. Transplant‐related mortality (TRM) was defined as death due to ASCT‐related causes other than disease relapse. Relapse‐free survival (RFS) was defined as the time from stem cell infusion to relapse or death from any cause. Overall survival (OS) was defined as the time from stem cell infusion to death of any cause. If patients survived without progression or death, survival was censored at the latest follow‐up date. Neutrophil engraftment was defined as an absolute neutrophil count (ANC) > 0.5 × 10^3^/μL on 3 consecutive measurements. Platelet (PLT) recovery was defined as seven consecutive measurements of 20 × 10^3^/μL without transfusion (TF). Response to ASCT was assessed at 3 months after ASCT as per the European Group for Blood and Bone Marrow Transplantation (EBMT) criteria.^13^

For all statistical analyses of effective variables, two‐tailed tests were performed. *P‐*values of <0.05 were considered statistically significant. All data were analyzed using the Statistical Package for the Social Sciences software (IBM® SPSS®Statistics, version 22.0).

## 3 RESULTS

### 3.1 Patient characteristics

From November 2011 to January 2020, 99 patients were enrolled. However, one patient received filgrastim instead of lenograstim and was excluded from the final analyses. At diagnosis, the median age was 59.0 years (range 35–70). Cytogenetics information was available from 77 of 98 patients (78.6%), and 30 of 77 (39.0%) patients were identified as a high‐risk group. The median laboratory findings at diagnosis were as follows: white blood cell (WBC) 5430 × 10^3^/μL, hemoglobin (Hb) 9.6 g/dL, PLT 211 × 10^3^/μL. The median number of previous lines of therapy before mobilization was 1 (range 1–4). Detailed description of induction chemotherapy before ASCT is indicated in Table S1. The number of patients exposed to lenalidomide and alkylator before mobilization was 6 (6.1%) and 11 (11.2%), respectively. Seven patients received radiation therapy (RT) before mobilization, including one patient undergoing RT to the pelvis area due to extramedullary multiple myeloma. The median body surface area (BSA) and body weight at the time of mobilization were 62.9 kg (range 35.9–104.5) and 1.67 m^2^ (range 1.20–2.20), respectively. Baseline patient characteristics are summarized in Table 1.

**TABLE 1:** Baseline characteristics of all patients.

Characteristics	*N* = 98
Age at diagnosis, years (median, range)	59.0 (35–70)
Male of patients, *N* (%)	45 (45.9%)
International Scoring System stage	
1 / 2 / 3 / Unknown	32 (32.7%) / 39 (39.8%) / 19 (19.4%) / 8 (8.2%)
Revised International Scoring System stage	
1 / 2 / 3 / Unknown	15 (15.3%) / 52 (53.1%) / 12 (12.2%) / 19 (19.4%)
Heavy chain type, *N* (%)	
IgG/A/D	50 (51.0%) / 20 (20.4%) / 2 (2.0%)
Light chain disease	23 (23.5%)
Unknown	3 (3.1%)
Light chain type, *N* (%)	
Kappa / Lambda / Unknown	56 (57.1%) / 40 (40.8%) / 2 (2.0%)
Lytic bone lesion >3	25 (25.5%)
Presence of plasmacytoma at diagnosis	13 (13.3%)
Laboratory finding at diagnosis	
Hemoglobin (median, range, g/dL)	9.6 (3.4–16.3)
WBC (median, range, 10^3^/μL)	5.43 (2.62–23.10)
Platelet (median, range, 10^3^/μL)	211 (58–532)
Cytogenetic abnormalities	*N* = 77
High risk	30 (39.0%)
Standard risk	47 (61.0%)
Lenalidomide exposure before mobilization, *N* (%)	6 (6.1%)
Alkylator exposure before mobilization, *N* (%)	11 (11.2%)
Radiotherapy before mobilization, *N* (%)	16 (16.3%)
Lines of therapy before mobilization, median (range)	1 (1–4)
BSA at mobilization, m^2^ (median, range)	1.67 (1.20–2.20)
Body weight at mobilization, kg (median, range)	62.9 (35.9–104.5)

Abbreviations: BSA, Body surface area; WBC, White blood cell.

### 3.2 Outcomes of mobilization and harvest apheresis

The median time from diagnosis to mobilization was 5 months (range 2.50–36.82), and all patients showed either PR or better response before mobilization (Table 2). The median number of apheresis was 3 (range 1–6). Most of patients (*N* = 90/98, 91.8%) underwent adequate mobilization (≥2 × 10^6^ CD34+ cells/kg body weight), while 58.2% (*N* = 57/98) underwent optimal mobilization (≥5 × 10^6^ CD34+ cells/kg body weight). All in all, the median CD34+ cells collected was 5.25 × 10^6^/kg (range 0.49–13.47) (Table 2).

**TABLE 2:** Outcomes of mobilization and harvest apheresis.

	*N* = 98 (%)
Time from diagnosis to mobilization, moths (range)	5.00 (2.50–36.82)
Disease status at mobilization	
PR	65 (66.3%)
VGPR	22 (22.4%)
CR	9 (9.2%)
sCR	2 (2.0%)
Median no. of apheresis procedures (median, range)	3 (1–6)
Median of total collected PB CD34+ cells using lenograstim only (median, range, × 10^6^/kg)	5.25 (0.49–13.47)
No. of patients collected PB CD34+ cells ≥2 × 10^6^/kg, *N* (%)	90 (91.8%)
No. of patients collected PB CD34+ cell ≥5 × 10^6^/kg, *N* (%)	57 (58.2%)
Additional modes of mobilization in mobilization failure patients, *N* (%)	*N* = 8
Plerixafor	7 (87.5%)
Cyclophosphamide	1 (12.5%)
Adverse events during mobilization	
Transfusion^a^	53 (54.1%)
RBC/PLT	5 (5.1%) / 51 (52.0%)
Bone pain^b^	6 (6.1%)
Fever	2 (2.0%)
GI trouble (nausea, diarrhea, abdominal pain)	2 (2.0%)

Abbreviations: CR, Complete response; GI, Gastrointestinal; G‐CSF, Granulocyte colony‐stimulating factor; PB, Peripheral blood; PLT, Platelet; PR, Partial response; RBC, Red blood cell; sCR, Stringent complete response; VGPR, Very good partial response.

^a^ Three patients received both RBC and PLT transfusions were calculated for each variable.

^b^ One patient with both bone pain and fever calculated for each variable.

**FIGURE 1:**
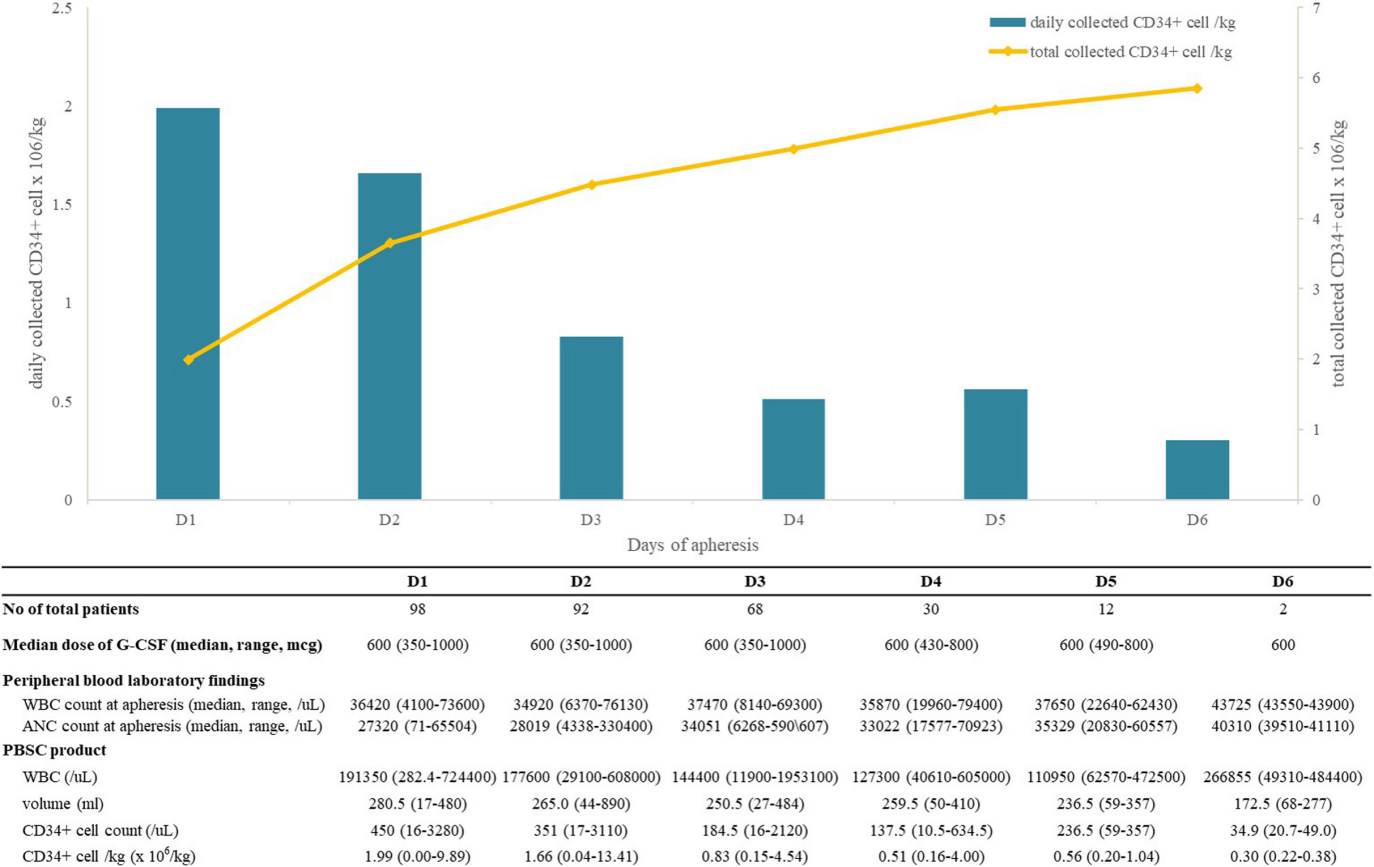
Daily CD34+ cell counts and hemogram changes during harvest apheresis following lenograstim mobilization. ANC, Absolute neutrophil count; G‐CSF, Granulocyte colony‐stimulating factor; PBSC, Peripheral blood stem cell; and WBC, White blood cell.

Changes in daily and total CD34+ cells collected after starting apheresis are detailed in Figure 1. Notably, 48.0% (*N* = 47/98) of the patients reached adequate mobilization with single apheresis. The median number of apheresis required for optimal collection of 5 × 10^6^ CD34 + cells/kg was 2 (range 1–5). Half of the patients (*N* = 49, 50.0%) showed optimal collection success within the third apheresis.

The mobilization failure rate with lenograstim (i.e., < 2 × 10^6^ CD34+ cells/kg body weight) was 8.2% (*N* = 8). Eight patients used additional drugs for mobilization: one patient with cyclophosphamide and seven patients with plerixafor. The median age of these patients at diagnosis was 57.5 years (range 47–65), and the median number of previous lines of therapy before mobilization was 1 (range 1–2). Only one patient was exposed to an alkylator before mobilization. None of the patients had previous radiation history. Further details of these patients are presented in Table S2.

### 3.3 Adverse events

Table 2 shows adverse events during lenograstim mobilization and harvest apheresis. During the procedure, 51 (52.0%) patients received PLT TF and 5 (5.1%) patients received red blood cell (RBC) TF. Other adverse events included bone pain (*N* = 6, 6.1%), gastrointestinal trouble (*N* = 2, 2.0%), and fever (*N* = 2, 2.0%).

### 3.4 Outcome of transplantation

This analysis was based on 94 patients who subsequently underwent ASCT (Figure S2). Four patients did not undergo ASCT after mobilization: death (*N* = 1), insufficient CD34+ cells collection (*N* = 1), and two patients were not subjected to ASCT up until data cutoff date. One case of death was due to a traffic accident after mobilization.

Of the 94 patients with ASCT, 77 (81.9%) underwent high‐dose melphalan conditioning, and 13 (13.8%) patients underwent busulfan‐melphalan conditioning. The median infused cell dose was 3.48 (range 1.80–11.00) × CD34+ cell/kg body weight. Neutrophil engraftment was observed in 93 (98.9%) patients, and platelet engraftment was observed in 89 (95.7%) patients. The median time to neutrophil and platelet engraftment was 10 days (range 1–21) and 10 days (range 1–37), respectively. TRM was observed in 1 patient; a 64‐year‐old patient expired 14 days after ASCT due to septic shock and clostridium difficile infection while awaiting cytopenia recovery. Regarding post‐ASCT response, 6.0% (5/84) patients achieved stringent complete response, 65.5% (55/84) complete response, 11.9% (10/84) very good partial response, and 15.5% (13/84) partial response. When following up hemogram every 3 months until 1 year after ASCT, hematologic recovery was verified and sustained within 12 months (Table S3).

Comparing the transplantation outcome according to the number of chemotherapy lines before ASCT (ASCT after 1st line of chemotherapy vs. ASCT after more than two lines of chemotherapy), there was no difference between neutrophil and platelet engraftment (Tables S4 and S5). Hematologic recovery was also identified and maintained until 12 months, with no significant differences between the two groups. Post‐ASCT responses showed a statistical difference between the two groups (*P* = 0.002); The response rate of more than complete response was higher in patients with ASCT after the first line of induction chemotherapy (77.1% vs. 42.9%).

During the median follow‐up of 33.47 months, the median RFS was 16.52 months (range 4.25–72.35), and the median OS was 31.04 months (range 1.25–107.18) for transplanted patients (Table 3). Relapse was observed in 35 patients (37.2%) over the entire follow‐up duration.

**TABLE 3:** Autologous stem cell transplantation (ASCT) outcomes.

	*N* (%)
Median age at ASCT, years (median, range)	58.0 (34–70)
Conditioning Regimen, N (%)	
HD‐Melphalan / Bu‐Mel / Others	77 (81.9%) / 13 (13.8%) / 4 (4.3%)
Time from diagnosis to ASCT, months (median, range)	6.23 (3.57–38.21)
Time from mobilization to ASCT, days (median, range)	34.0 (11.0–183.0)
Infused CD34 cell, ×10^6^/kg (median)	3.48 (1.80–11.00)
Neutrophil engraftment	93 (98.9%)
Time to neutrophil engraftment, days (median, range)	10 (1–21)
Platelet recovery	89 (94.7%)
Time to platelet recovery, days (median, range)	10 (1–37)
Relapse after ASCT for follow‐up duration	35 (37.6%)
Median relapse free survival	16.52 (4.25–72.35)
Median overall survival (from ASCT)	31.04 (1.25–107.18)
Post ASCT response	*N* = 84
Relapse	1 (1.2%)
PR	13 (15.5%)
VGPR	10 (11.9%)
CR	55 (65.5%)
sCR	5 (6.0%)

Abbreviations: ASCT, Autologous stem cell transplantation; Bu, Busulfan; CR, Complete response; HD, High‐dose; PR, Partial response; sCR, Stringent complete response; VGPR, Very good partial response.

### 3.5 Variables associated with mobilization

In the multiple linear regression model, prognostic factors significantly related to the total collected CD34+ cell yield were BSA at mobilization and PLT transfusion during apheresis (Table S6). The BSA at mobilization was positively correlated with CD34+ cell yield, whereas PLT transfusion during cell collection showed a negative correlation with CD34+ cell yield.

## 4 DISCUSSION

As the discussion of glycosylated G‐CSF mobilization efficacy and safety is ongoing and a consensus has not been reached, we conducted this prospective trial. We found that (1) 91.8% succeeded in collecting an adequate quantity of stem cells and 58.2% in collecting optimal quantity within three aphereses, (2) there were no significant safety issues during mobilization, (3) the quality of the collected stem cells was satisfactory as evident by high engraftment rates and stable post‐ASCT hemograms.

Successful mobilization of PBSC is directly related to favorable transplant outcomes and longer survivals.^14^, ^15^ However, repeated rounds of apheresis to acquire an optimal amount of stem cells can induce economic burdens and increase the risk of complications while compromising stem cell quality. As such, several mobilization regimens have been suggested over the years. Coinciding with more recent studies reporting similar efficacies of glycosylated G‐CSF versus nonglycosylated G‐CSF mobilization,^6^, ^7^, ^16^, ^17^ our study prospectively demonstrated the feasibility of glycosylated G‐CSF mobilization. In particular, our results were considered to be comparable to filgrastim, which is recommended for mobilization in the recent guideline. A prior study demonstrated no differences in mobilization efficacy among biosimilar filgrastim, original filgrastim, and lenograstim and no mobilization failure in all three groups.^18^ Meanwhile, *Roberto* et al. reported 47% of patients with filgrastim for minimal collection in lymphoproliferative disease,^7^ and 80.3% of patients were identified to attain minimal collection in MM with filgrastim only.^19^ In our study, 91.8% of patients fulfilled minimal collection doses, and only one pheresis was needed to obtain an adequate cell dose in 48% of the enrolled patients. The mobilization failure rate was only 8.2%, which is also comparable with (6.3%–26%) reported in previous studies.^9^, ^20^, ^21^, ^22^, ^23^, ^24^

In order to minimize the mobilization failure rate, we investigated the predictive factors for poor mobilization. Risk factors for harvest failure include age,^25^, ^26^, ^27^ previous lenalidomide or alkylator exposure,^28^, ^29^, ^30^, ^31^ extensive treatment course, and radiation history before ASCT.^26^, ^32^ Interestingly, age was not predictive of poor mobilization in our study. One‐third of patients enrolled in this study were ≥ 60 years old: 53.8% attained optimal collection, and mobilization failure occurred in only one patient (Table S7). Although the median number of apheresis required for optimal collection was bigger in elderly patients (age ≤ 60, 2 vs. age > 60, 3, *P* = 0.045), overall lenograstim attained successful mobilization and engraftment with a relatively tolerable safety profiles in this population. As MM is a disease of the elderly,^33^ the utility of lenograstim mobilization in elderly patients holds clinical significance.

On the contrary, we recognized PLT TF resulting from thrombocytopenia during PBSC mobilization as an associated factor influencing optimal harvest. Steady‐state thrombocytopenia at baseline and at the time of mobilization is deemed as a risk factor associated with poor mobilization.^14^ Indeed, thrombocytopenia at the time of mobilization is an established predictive marker of mobilization failure in chemotherapy‐based regimens.^34^, ^35^ Whether PLT TF or thrombocytopenia is also a predictive marker in G‐CSF only mobilization remains elusive, but we hereby provide evidence that it might indeed be.

Equally important is the quality of the harvested stem cells. In our study, most of the patients showed engraftment of neutrophils (98.9%) and PLT (94.7%), consistent with the results of previous trials conducted on mobilization in MM.^19^, ^36^ Concerning response after ASCT, several studies showed different results regarding the post ASCT response; *Tuchman* et al. reported that 7% of patients obtained CR with G‐CSF only mobilization,^36^ and Bacon et al.'s results presented that 60% of patients achieved a VGPR or better after ASCT with G‐CSF only mobilization group in MM.^37^ In our study of post‐ASCT response, 71.4% (60/84) and 83.3% (70/84) of patients acquired CR and VGPR or better, respectively. It is plausible to hypothesize that the acceptable transplantation outcome of our study resulted from the excellent quality of the mobilized stem cells. Moreover, as shown in the hemogram follow‐up in Table S3, hematologic recovery was stably maintained after 1 year, supporting the quality of stem cells of lenograstim only mobilization. One of the strengths of our study is that we provide information on the quality of the harvested stem cells based on ASCT results and post‐ASCT sequential hemograms.

The limitation of our study is a center‐to‐center variation of the treatment scheme of myeloma, including induction therapy, mobilization, and ASCT procedures. As such, there may be a confounder for patients underwent mobilization. Thus, our results should be cautiously interpreted. Subsequent large trials are needed to confirm the efficacy and identify the risk factor for failure or suboptimal mobilization with more homogeneous patients.

In conclusion, glycosylated G‐CSF can also be used for the mobilization of stem cells in MM patients, regardless of patients' age. The efficacy and safety profiles of lenograstim mobilization are comparable with previous nonglycosylated G‐CSF mobilization data.

## AUTHOR CONTRIBUTIONS

Contribution: Sung‐Soo Yoon conceptualized and designed the study. Eun Hee Jung, Ja Min Byun, and Sung‐Soo Yoon performed formal analysis and writing‐original draft preparation. All authors were involved in the investigation, data curation, and contributed to manuscript revision.

## CONFLICT OF INTEREST STATEMENT

None.

## ETHICS STATEMENT

This study was conducted according to the Declaration of Helsinki and was approved by the Institutional Review Board of each participating hospital (Seoul National University Hospital, Ulsan University Hospital, Busan Paik Hospital, Keimyung University Dongsan Medical Center, and Korea Cancer Center Hospital). Informed consent was taken from all patients before participating in any study‐related procedure.

## Supporting information


Data S1.

## Data Availability

The data that support the findings of this study are available upon reasonable request from the corresponding author.
