# Supplementary material for: Mobilization of hematopoietic stem cells with lenograstim in multiple myeloma patients: Prospective multicenter observational study (KMM122)
Source: Cancer Med. 2023 Mar 23;12(8):9186–93. doi: 10.1002/cam4.5657 (PMC10166964; doi:10.1002/cam4.5657)
Supplement: Supplementary file 1 — Data S1. [file CAM4-12-9186-s001.docx]

**Supplementary Materials**

**Index**

Supplementary Table 1 ------------------------------------------------------------------------- Page 2

Supplementary Table 2 ----------------------------------------------------------------------- Page 3-4

Supplementary Table 3 ------------------------------------------------------------------------- Page 5

Supplementary Table 4 ----------------------------------------------------------------------- Page 6-9

Supplementary Table 5 ------------------------------------------------------------------------ Page 10

Supplementary Table 6 ------------------------------------------------------------------------ Page 11

Supplementary Table 7 ---------------------------------------------------------------------- Page 12-14

Supplementary Figure 1 ----------------------------------------------------------------------- Page 15

Supplementary Figure 2 ----------------------------------------------------------------------- Page 16

| **Supplementary Table 1.** Details of induction chemotherapy of patients | | |  |
| --- | --- | --- | --- |
| **Variables** |  | **N (%)** |  |
| **Mobilization after 1st line (N = 82)** |  |  |  |
| Without alkylating agent |  |  |  |
| TD |  | 19 (22.9%) |  |
| VD |  | 5 (6.0%) |  |
| VTD |  | 50 (60.2%) |  |
| Others |  | 2 (2.4%) |  |
| With alkylating agent |  |  |  |
| VMP |  | 1 (1.2%) |  |
| TCD |  | 3 (3.6%) |  |
| VCD |  | 2 (2.4%) |  |
| **Mobilization after ≥2nd line (N = 16)** |  |  |  |
| after 2nd line (N = 13) |  |  |  |
| Without alkylating agent during induction chemotherapy |  | 9 (56.3%) |  |
| With alkylating agent during induction chemotherapy |  | 4 (25.0%) |  |
| after ≥ 3rd line (N = 3) |  |  |  |
| Without alkylating agent during induction chemotherapy |  | 2 (12.5%) |  |
| With alkylating agent during induction chemotherapy |  | 1 (6.3%) |  |

TD, thalidomide-dexamethasone; VD, bortezomib-dexamethasone; VTD, bortezomib-thalidomide-dexamethasone; VMP, bortezomiab-melphalan-prednisolon; TCD, thalidomide-cyclophosphamide-dexamethasone; VCD, bortezomib-cyclophosphamide-dexamethasone.

**Supplementary Table 2.** Details of mobilization failure patients

|  | **1** | **2** | **3** | **4** | **5** | **6** | **7** | **8** |
| --- | --- | --- | --- | --- | --- | --- | --- | --- |
| ***Baseline Characteristics*** |  |  |  |  |  |  |  |  |
| **Age at diagnosis / Sex** | 57 / F | 50 / F | 58 / F | 62 / F | 50 / F | 49 / M | 46 / M | 57 / F |
| **International Scoring System stage** | 3 | 2 | 2 | 2 | N/A | 2 | 2 | 2 |
| **Revised International Scoring System stage** | 3 | 2 | 2 | 2 | N/A | 2 | 2 | 2 |
| **Heavy chain type** | Light chain disease | Light chain disease | Light chain disease | IgG | Light chain disease | IgA | Light chain disease | IgG |
| **Light chain type** | Lambda | Lambda | Kappa | Lambda | Kappa | Lambda | Lambda | Lambda |
| **Lytic bone lesion > 3** | No | Yes | No | Yes | No | No | No | No |
| **Presence of plasmacytoma at diagnosis** | No | No | No | No | No | No | No | No |
| **Laboratory finding at diagnosis** |  |  |  |  |  |  |  |  |
| Hemoglobin (median, range, g/dL) | 11.3 | 6.2 | 11.3 | 9.2 | Unknown | 5.5 | 9.4 | 5.7 |
| WBC (median, range, 10^3^/μL) | 4.04 | 9.62 | 4.49 | 3.9 | Unknown | 5.7 | 6.88 | 3.83 |
| Platelet (median, range, 10^3^/μL) | 170 | 186 | 365 | 198 | Unknown | 64 | 188 | 140 |
| **Cytogenetic abnormalities** | Standard | Standard | Unknown | Standard | High | High | Standard | Standard |
| **Lenalidomide exposure before mobilization** | No | No | No | No | Yes | No | No | No |
| **Alkylator exposure before mobilization** | No | No | Yes | No | No | No | No | No |
| **Radiotherapy before mobilization** | No | No | No | No | No | No | No | No |
| **Lines of therapy before mobilization, median** | 1 | 1 | 2 | 1 | 2 | 1 | 1 | 1 |
| ***Mobilization & Apheresis*** |  |  |  |  |  |  |  |  |
| **BSA at mobilization, m^2^** | 1.41 | 1.39 | 1.65 | 1.75 | 1.22 | 1.88 | 2.04 | 1.54 |
| **Body weight at mobilization, kg** | 46.80 | 49.45 | 65.65 | 69.40 | 40.25 | 75.90 | 89.20 | 53.70 |
| **Time from diagnosis to ASCT, months** | 8.79 | 6.00 | 11.11 | 5.50 | 16.00 | 4.96 | before ASCT | 7.18 |
| **Time from diagnosis to mobilization, months** | 5.89 | 4.57 | 9.89 | 4.57 | 9.46 | 3.61 | 6.21 | 6.57 |
| **Time from mobilization to ASCT, days** | 81.00 | 40.00 | 34.00 | 26.00 | 183.00 | 38.00 | before ASCT | 17.00 |
| **Disease status at mobilization** | CR | VGPR | PR | PR | PR | PR | PR | PR |
| **No. of apheresis procedures (median, range)** | 4 | 3 | 2 | 3 | 3 | 3 | 3 | 6 |
| **Total collected PB CD34+ cells using G-CSF only, x 10^6^/kg (median, range)** | 1.86 | 0.72 | 0.9 | 0.59 | 0.49 | 1.99 | 1.61 | 1.65 |
| **Additional modes of mobilization** | Plerixafor | Plerixafor | Cyclophosphamide | Plerixafor | Plerixafor | Plerixafor | Plerixafor | Plerixafor |
| **Total collected PB CD34+ cells with additional modes of mobilization** | 3.51 | 4.15 | 4.43 | 2.16 | 2.18 | 4.69 | 3.31 | 2.64 |
| **No. of patients with transfusion during apheresis** |  |  |  |  |  |  |  |  |
| RBC | No | No | No | No | Yes | No | Yes | No |
| PLT | Yes | Yes | No | Yes | Yes | Yes | No | No |
| ***ASCT*** |  |  |  |  |  |  |  |  |
| **Conditioning Regimen** | Busulfan/Etoposide/ Cyclophosphamide | High-dose  melphalan | High-dose  melphalan | High-dose  melphalan | High-dose  melphalan | Busulfan/Melphalan | before ASCT | High-dose  melphalan |
| **Infused CD34 cell, ×10^6^/kg (median)** | 2.717 | 4.15 | 4.43 | 2.16 | 2.069 | 2.32 |  | 2.64 |
| **Neutrophil engraftment, days** | Yes (9) | Yes (9) | Yes (9) | Yes (13) | Yes (11) | Yes (11) |  | Yes (12) |
| **Platelet recovery** | Yes (11) | Yes (8) | Yes (8) | Yes (11) | Yes (12) | Yes (6) |  | Yes (20) |
| **Post ASCT response** | CR | CR | CR | CR | CR | CR |  | CR |
| **Relapse after ASCT** | No | No | No | No | Yes | Yes |  | No |
| **Median relapse free survival** |  |  |  |  | 5.68 | 17.00 |  |  |
| **Median overall survival (from ASCT)** | 43.93 | 42.36 | 28.79 | 32.36 | 14 | 17 |  | 51.21 |

F, female; M, male; WBC, white blood cell; ASCT, autologous stem cell transplantation; BSA, body surface area; G-CSF, granulocyte colony stimulating factor; RBC; red blood cell; PLT, platelet

**Supplementary Table 3.** Hematologic recovery within 12 months after autologous stem cell transplantation

|  | **3 months** | **6 months** | **9 months** | **12 months** |
| --- | --- | --- | --- | --- |
| **WBC (median, range, 10^3^/μL)** | 4.60 (1.79-38.31) | 4.86 (2.09-28.47) | 5.00 (1.03-29.55) | 5.10 (1.83-34.37) |
| **ANC (median, range, 10^3^/μL)** | 2.01 (0.35-7.90) | 2.29 (0.37-7.42) | 2.27 (0.40-7.77) | 2.46 (0.45-13.08) |
| **Hb (median, range, g/dL)** | 11.6 (7.0-14.9) | 12.1 (8.2-15.1) | 12.4 (8.7-15.5) | 12.6 (8.7-15.8) |
| **PLT (median, range, 10^3^/μL)** | 147 (14-269) | 168 (35-320) | 162 (13-564) | 168 (21-278) |

WBC, white blood cell; ANC, absolute neutrophil count; Hb, hemoglobin; PLT, platelet

| **Supplementary Table 4.** Details of characteristics of patients according to number of induction therapy | | | |  |  | | |  |  |
| --- | --- | --- | --- | --- | --- | --- | --- | --- | --- |
| **Characteristics** | **ASCT after 1st line of induction therapy  (N = 82)** |  | **ASCT after ≥ 2 line of induction therapy (N = 16)** | | |  | ***p*-value** | |  |
|  |  |  |  |  |  |  |  |  |  |
| **Age at diagnosis, years (median, range)** | 57.0 (33-66) |  | 58.0 (43-69) | | |  | 0.308 | |  |
| **Male of patients, N (%)** | 40 (48.8%) |  | 5 (33.3%) | | |  | 0.400 | |  |
| **International Scoring System stage** |  |  |  | | |  | 0.967 | |  |
| 1 / 2 / 3 / Unknown | 28 (34.1%) / 31 (37.8%) / 16 (19.5%) / 7 (8.5%) |  | 4 (26.7%) / 8 (55.3%) / 2 (13.3%) / 1 (6.7%) | | |  |  | |  |
| **Revised International Scoring System stage** |  |  |  | | |  | 0.816 | |  |
| 1 / 2 / 3 / Unknown | 14 (17.1%) / 42 (51.2%) / 10 (12.2%) / 16 (19.5%) |  | 1 (6.7%) / 10 (66.7%) / 1 (6.7%) / 3 (20.0%) | | |  |  | |  |
| **Heavy chain type, N (%)** |  |  |  | | |  | 0.193 | |  |
| IgG/A/D | 40 (48.2%) / 19 (22.9%) / 2 (2.4%) |  | 10 (66.7%) / 1 (6.7%) / 0 (0.0%) | | |  |  | |  |
| Light chain disease | 19 (22.9%) |  | 4 (26.7%) | | |  |  | |  |
| Unknown | 3 (3.6%) |  | 0 (0.0%) | | |  |  | |  |
| **Light chain type, N (%)** |  |  |  | | |  | 0.003 | |  |
| Kappa / Lambda / Unknown | 42 (50.6%) / 39 (47.0%) / 2 (2.4%) |  | 14 (93.3%) / 1 (6.7%) / 0 (0.0%) | | |  |  | |  |
| **Lytic bone lesion > 3** | 23 (27.7%) |  | 2 (13.3%) | | |  | 0.249 | |  |
| **Presence of plasmacytoma at diagnosis** | 10 (12.0%) |  | 3 (20.0%) | | |  | 0.688 | |  |
| **Laboratory finding at diagnosis** |  |  |  | | |  |  | |  |
| Hemoglobin (median, range, g/dL) | 9.4 (3.4-15.2) |  | 10.5 (8.3-16.3) | | |  | 0.028 | |  |
| WBC (median, range, 10^3^/μL) | 5.39 (2.69-23.10) |  | 5.61 (2.62-9.12) | | |  | 0.801 | |  |
| Platelet (median, range, 10^3^/μL) | 205 (58-532) |  | 247 (153-365) | | |  | 0.278 | |  |
| **Cytogenetic abnormalities** | N = 66 |  | N = 11 | | |  | 0.245 | |  |
| High risk | 22 (26.5%) |  | 8 (72.7%) | | |  |  | |  |
| Standard risk | 44 (53.0%) |  | 3 (27.3%) | | |  |  | |  |
| **Lenalidomide exposure before mobilization, N (%)** | 0 (0.0%) |  | 6 (40.0%) | | |  | <0.001 | |  |
| **Alkylator exposure before mobilization, N (%)** | 6 (7.2%) |  | 2 (13.3%) | | |  | 0.604 | |  |
| **BSA at mobilization, m^2^ (median, range)** | 1.68 (1.20-2.20) |  | 1.65 (1.22-2.03) | | |  | 0.336 | |  |
| **Body weight at mobilization, kg (median, range)** | 63.2 (35.9-104.5) |  | 61.2 (40.3-85.2) | | |  | 0.438 | |  |
| ***Mobilization*** |  |  |  | | |  |  | |  |
| **Time from diagnosis to mobilization, moths (range)** | 4.75 (2.50-17.79) |  | 7.96 (4.57-36.82) | | |  | <0.001 | |  |
| **Disease status at mobilization** |  |  |  | | |  | 0.397 | |  |
| PR | 54 (65.1%) |  | 11 (73.3%) | | |  |  | |  |
| VGPR | 18 (21.7%) |  | 4 (26.7%) | | |  |  | |  |
| CR | 9 (10.8%) |  | 0 (0.0%) | | |  |  | |  |
| sCR | 2 (2.4%) |  | 0 (0.0%) | | |  |  | |  |
| **D-4** |  |  |  | | |  |  | |  |
| WBC (median, range, 10^3^/μL) | 5.80 (2.92-46.40) |  | 5.90 (2.33-61.00) | | |  | 0.847 | |  |
| Hb (median, range, g/dL) | 12.4 (9.2-15.1) |  | 11.6 (7.3-15.1) | | |  | 0.319 | |  |
| PLT (median, range, 10^3^/μL) | 268 (103-473) |  | 229 (93-355) | | |  | 0.286 | |  |
| **D-3** |  |  |  | | |  |  | |  |
| WBC (median, range, 10^3^/μL) | 18.15 (5.26-44.43) |  | 27.00 (3.97-41.70) | | |  | 0.032 | |  |
| Hb (median, range, g/dL) | 12.0 (8.6-14.8) |  | 12.6 (8.6-15.8) | | |  | 0.742 | |  |
| PLT (median, range, 10^3^/μL) | 237 (67-445) |  | 230 (90-336) | | |  | 0.712 | |  |
| **D-2** |  |  |  | | |  |  | |  |
| WBC (median, range, 10^3^/μL) | 24.20 (13.39-74.95) |  | 36.88 (4.09-59.70) | | |  | 0.031 | |  |
| Hb (median, range, g/dL) | 11.9 (8.8-14.6) |  | 12.3 (9.1-14.6) | | |  | 0.827 | |  |
| PLT (median, range, 10^3^/μL) | 227 (79-385) |  | 252 (80-370) | | |  | 0.629 | |  |
| **D-1** |  |  |  | | |  |  | |  |
| WBC (median, range, 10^3^/μL) | 30.68 (1.19-75.71) |  | 37.42 (3.92-53.51) | | |  | 0.403 | |  |
| Hb (median, range, g/dL) | 12.0 (8.2-14.2) |  | 11.7 (8.3-15.3) | | |  | 0.717 | |  |
| PLT (median, range, 10^3^/μL) | 208 (59-370) |  | 221 (65-318) | | |  | 0.681 | |  |
| ***Apheresis*** |  |  |  | | |  |  | |  |
| **Median no. of apheresis procedures** | 3 (1-6) |  | 3 (1-5) | | |  | 0.639 | |  |
| **Median of total collected PB CD34+ cells using G-CSF only (x 10^6^/kg)** | 5.40 (0.59-13.47) |  | 4.16 (0.49-10.42) | | |  | 0.135 | |  |
| **No. of patients collected PB CD34+ cells ≥ 2 x 10^6^/kg** | 77 (92.8%) |  | 13 (86.7%) | | |  | 0.604 | |  |
| **No. of patients collected PB CD34+ cell ≥ 5 x 10^6^/kg** | 51 (61.4%) |  | 6 (40.0%) | | |  | 0.158 | |  |
| **Median no. of apheresis required for minimal collection** | 1 (1-5) |  | 1 (1-4) | | |  | 0.974 | |  |
| **Median no. of apheresis required for optimal collection** | 2 (1-5) |  | 3 (1-4) | | |  | 0.402 | |  |
| **Use of other drugs for mobilization, n (%)** | N = 6 |  | N = 2 | | |  | 1.000 | |  |
| Cyclophosphamide | 1 (16.7%) |  | 0 (0.0%) | | |  |  | |  |
| plerixafor | 5 (83.3%) |  | 2 (100.0%) | | |  |  | |  |
| **PBSC product ; CD34+ cell /kg (x 10^6^/kg)** |  |  |  | | |  |  | |  |
| D1 | N = 83 |  | N = 15 | | |  |  | |  |
|  | 2.00 (0.06-9.89) |  | 1.97 (0.00-5.15) | | |  | 0.586 | |  |
| D2 | N = 78 |  | N = 14 | | |  |  | |  |
|  | 1.75 (0.04-13.41) |  | 1.29 (0.10-5.27) | | |  | 0.077 | |  |
| D3 | N = 55 |  | N = 12 | | |  |  | |  |
|  | 0.90 (0.15-4.54) |  | 0.48 (0.10-2.34) | | |  | 0.192 | |  |
| D4 | N = 25 |  | N = 5 | | |  |  | |  |
|  | 0.50 (0.16-1.23) |  | 0.70 (0.39-4.00) | | |  | 0.474 | |  |
| D5 | N = 11 |  | N = 1 | | |  |  | |  |
|  | 0.55 (0.20-1.04) |  | 0.86 | | |  | 0.333 | |  |
| ***ASCT*** |  |  |  | | |  |  | |  |
| **Time from diagnosis to ASCT, months (median, range)** | 5.93 (3.57-19.68) |  | 8.43 (5.50-38.21) | | |  | <0.001 | |  |
| **Time from mobilization to ASCT, days (median, range)** | 33.50 (11.00-93.00) |  | 35.50 (12.30-183.00) | | |  | 0.313 | |  |
| **Infused CD34 cell, ×10^6^/kg (median)** | 3.40 (1.80-11.00) |  | 3.82 (2.0-5.2) | | |  | 0.643 | |  |
| **Neutrophil engraftment** | 78 (78/79, 98.7%) |  | 15 (15/15, 100.0%) | | |  | 0.516 | |  |
| Time to neutrophil engraftment, days (median, range) | 10 (1-21) |  | 10 (9-14) | | |  | 0.333 | |  |
| **Platelet engraftment** | 74 (74/78, 94.9%) |  | 15 (15/15, 100.0%) | | |  | 0.599 | |  |
| Time to platelet recovery, days (median, range) | 10 (1-33) |  | 11 (5-37) | | |  | 0.452 | |  |
| **Relapse after ASCT for follow-up duration** | 27 (32.5%) |  | 9 (60.0%) | | |  | 0.083 | |  |
| **Median relapse free survival** | 18.43 (6.57-72.35) |  | 9.75 (4.25-52.67) | | |  | 0.043 | |  |
| **Median overall survival (from ASCT)** | 31.11 (0.50-107.18) |  | 29.41 (4.00-85.07) | | |  | 0.620 | |  |
| **Post ASCT response** | N = 70 |  | N = 14 | | |  | 0.002 | |  |
| Relapse | 0 (0.0%) |  | 1 (7.1%) | | |  |  | |  |
| PR | 7 (10.0%) |  | 6 (42.9%) | | |  |  | |  |
| VGPR | 9 (12.9%) |  | 1 (7.1%) | | |  |  | |  |
| CR | 49 (70.0%) |  | 6 (42.9%) | | |  |  | |  |
| sCR | 5 (7.1%) |  | 0 (0.0%) | | |  |  | |  |

ASCT, autologous stem cell transplantation; BSA, body surface area, CR, Complete response; G-CSF, granulocyte colony stimulating factor; Hb, hemoglobin; PLT, platelet; PB, peripheral blood; PBSC, peripheral blood stem cell; PR, Partial response; sCR, Stringent complete response; and VGPR, Very good partial response; and WBC, white blood cell.

| **Supplementary Table 5.** Hematologic recovery within 12 months after autologous stem cell transplantation according to the number of lines of induction chemotherapy | | | | | | | | | | | | | | | |
| --- | --- | --- | --- | --- | --- | --- | --- | --- | --- | --- | --- | --- | --- | --- | --- |
|  | **3 months** | | | | **6 months** | | | | **9 months** | | | | **12 months** | | |
|  | | 1st line | ≥ 2 line | p-value | | 1st line | ≥ 2 line | p-value | | 1st line | ≥ 2 line | p-value | 1st line | ≥ 2 line | p-value |
| **WBC (median, range, 10^3^/μL)** | | 4.43 (1.79-38.31) | 5.57 (3.22-6.30) | 0.115 | | 4.71 (2.09-28.47) | 5.21 (3.00-9.56) | 0.548 | | 5.41 (1.03-29.55) | 5.00 (1.91-8.87) | 0.750 | 5.71 (1.83-34.37) | 4.86 (3.77-7.80) | 0.853 |
| **ANC (median, range, 10^3^/μL)** | | 1.88 (0.35-7.90) | 2.37 (0.05-3.63) | 0.230 | | 2.37 (0.68-7.42) | 2.55 (1.62-5.62) | 0.610 | | 2.33 (0.18-7.77) | 2.63 (0.84-6.18) | 0.556 | 2.58 (0.67-13.08) | 2.77 (1.23-5.22) | 0.794 |
| **Hb (median, range, g/dL)** | | 12.0 (7.0-14.9) | 11.4 (10.0-14.6) | 0.186 | | 12.4 (9.7-15.1) | 11.7 (8.2-15.0) | 0.092 | | 12.5 (9.7-15.5) | 11.9 (8.7-14.9) | 0.087 | 12.8 (9.4-15.4) | 11.9 (8.7-15.8) | 0.269 |
| **PLT (median, range, 10^3^/μL)** | | 142 (14-269) | 140 (68-241) | 0.301 | | 167 (35-320) | 179 (58-288) | 0.193 | | 163 (29-564) | 157 (13-243) | 0.788 | 170 (21-269) | 152 (55-278) | 0.194 |

WBC, white blood cell; ANC, absolute neutrophil count; Hb, hemoglobin; PLT, platelet

**Supplementary Table 6.** Multiple linear regression model of variables affecting collected PB CD34+ cells with G-CSF only

|  |  | **Coefficient** | **Standard Error** | **t** | **P-value** |
| --- | --- | --- | --- | --- | --- |
| **BSA at mobilization** |  | 3.336 | 1.603 | 2.080 | 0.040 |
| **PLT TF during PBSCM** |  | -1.472 | 0.593 | -2.483 | 0.015 |

PB, peripheral blood; G-CSF, granulocyte colony stimulating factor; BSA, body surface area; PLT, platelet; TF, transfusion; PBSCM, peripheral blood stem cell mobilization

**Supplementary Table 7.** Details of patients according to age at ASCT

|  | **Age ≤ 60 (N = 67)** | **Age > 60 (N = 27)** | ***p*-value** |
| --- | --- | --- | --- |
| ***Baseline characteristics*** |  |  |  |
| **Age at diagnosis (median, years)** | 53.5 (33-59) | 62.0 (58-69) | <0.001 |
| **Male of patients, n (%)** | 31 (46.3%) | 12 (44.4%) | 0.873 |
| **International Scoring System stage** |  |  | 0.898 |
| 1 / 2 / 3 / Unknown | 23 (34.3%) / 26 (38.8%) / 11 (16.4%) / 7 (10.4%) | 9 (33.3%) / 11 (40.7%) / 6 (22.2%) / 1 (3.7%) |  |
| **Revised International Scoring System stage** |  |  | 0.881 |
| 1 / 2 / 3 / Unknown | 12 (17.9%) / 33 (49.3%) / 8 (11.9%) / 14 (20.9%) | 3 (11.1%) / 16 (59.3%) / 3 (11.5%) / 5 (18.5%) |  |
| **Heavy chain type, n (%)** |  |  | 0.679 |
| IgG/A/D | 33 (49.3%) / 13 (19.4%) / 2 (3.0%) | 15 (55.6%) / 6 (22.2%) / 0 (0.0%) |  |
| Light chain disease | 17 (25.4%) | 5 (18.5%) |  |
| Unknown | 2 (3.0%) | 1 (3.7%) |  |
| **Light chain type, n (%)** |  |  | 0.201 |
| Kappa / Lambda / Unknown | 36 (53.7%) / 29 (43.3%) / 2 (3.0%) | 19 (70.4%) / 8 (29.6%) / 0 (0.0%) |  |
| **Lytic bone lesion > 3** | 17 (25.4%) | 7 (25.9%) | 0.896 |
| **Presence of plasmacytoma** | 11 (16.4%) | 2 (7.4%) | 0.194 |
| **Laboratory finding at diagnosis, median, range** |  |  |  |
| Hb (g/dL) | 9.8 (3.4-15.2) | 9.5 (6.9-16.3) | 0.974 |
| WBC (10^3^/μL) | 5.41 (2.62-23.10) | 5.24 (2.69-12.20) | 0.630 |
| PLT (10^3^/μL) | 217 (58-532) | 205 (84-358) | 0.329 |
| Serum β2-microglobulin (mg/dL) | 2.76 (1.55-16.73) | 3.49 (1.85-19.47) | 0.069 |
| BM plasma cell count (%) | 24.3 (0.2-85.8) | 31.8 (1.5-73.9) | 0.880 |
| **Cytogenetic abnormalities** | N = 53 | N = 21 | 0.881 |
| High risk / Standard risk | 20 (37.7%) / 33 (62.3%) | 9 (42.9%) / 12 (87.1%) |  |
| **Exposure before mobilization, N (%)** |  |  |  |
| Thalidomide | 62 (92.5%) | 21 (77.8%) | 0.045 |
| Lenalidomide | 3 (4.5%) | 3 (11.1%) | 0.236 |
| **Previous lines of treatment before ASCT** |  |  | 0.183 |
| 1 / 2 / ≥ 3 | 58 (86.6%) / 7 (10.4%) / 2 (3.0%) | 21 (77.8%) / 4 (14.8%) / 2 (7.4%) |  |
| **Previous radiation therapy before ASCT** | 5 (7.5%) | 1 (3.7%) | 0.502 |
| **Time from diagnosis to mobilization, months (median, range)** | 5.11 (2.5-36.82) | 4.81 (2.93-31.36) | 0.515 |
| ***Mobilization*** |  |  |  |
| **D-4** |  |  |  |
| WBC (median, range, 10^3^/μL) | 5.74 (2.33-46.40) | 6.50 (3.72-61.00) | 0.235 |
| Hb (median, range, g/dL) | 12.3 (7.3-14.9) | 11.4 (9.2-15.1) | 0.209 |
| PLT (median, range, 10^3^/μL) | 268 (93-473) | 258 (126-357) | 0.107 |
| **D-3** |  |  |  |
| WBC (median, range, 10^3^/μL) | 18.15 (3.97-44.43) | 20.05 (6.90-41.70) | 0.323 |
| Hb (median, range, g/dL) | 12.2 (8.6-14.8) | 11.8 (8.6-15.8) | 0.912 |
| PLT (median, range, 10^3^/μL) | 234 (67-445) | 233 (160-290) | 0.808 |
| **D-2** |  |  |  |
| WBC (median, range, 10^3^/μL) | 24.78 (4.09-49.47) | 26.12 (17.17-74.95) | 0.215 |
| Hb (median, range, g/dL) | 12.3 (8.8-14.6) | 11.7 (9.1-14.6) | 0.310 |
| PLT (median, range, 10^3^/μL) | 229 (79-385) | 231 (92-302) | 0.643 |
| **D-1** |  |  |  |
| WBC (median, range, 10^3^/μL) | 30.91 (3.92-75.71) | 31.83 (11.19-74.71) | 0.680 |
| Hb (median, range, g/dL) | 12.2 (8.6-14.2) | 11.7 (8.3-15.3) | 0.442 |
| PLT (median, range, 10^3^/μL) | 202 (59-370) | 229 (75-277) | 0.753 |
| ***Apheresis*** |  |  |  |
| **Median no. of apheresis procedures** | 3 (1-6) | 3 (2-5) | 0.217 |
| **Median of total collected PB CD34+ cells using G-CSF only (x 10^6^/kg)** | 5.58 (0.49-13.13) | 5.18 (0.59-13.47) | 0.247 |
| **No. of patients collected PB CD34+ cells ≥ 2 x 10^6^/kg** | 62 (92.5%) | 26 (96.3%) | 0.383 |
| **No. of patients collected PB CD34+ cell ≥ 5 x 10^6^/kg** | 42 (62.7%) | 15 (55.6%) | 0.524 |
| **Median no. of apheresis required for minimal collection** | 1 (1-5) | 2 (1-5) | 0.383 |
| **Median no. of apheresis required for optimal collection** | 2 (1-5) | 3 (2-5) | 0.524 |
| **No. of patients with transfusion during apheresis** |  |  |  |
| RBC | 3 (4.5%) | 1 (3.7%) | 0.867 |
| PLT | 36 (53.7%) | 14 (51.9%) | 0.869 |
| **Use of other drugs for mobilization, n (%)** |  |  | 0.720 |
| Cyclophosphamide | 1 (1.5%) | 0 (0.0%) |  |
| plerixafor | 6 (9.0%) | 1 (3.7%) |  |
| **PBSC product ; CD34+ cell /kg (x 10^6^/kg)** |  |  |  |
| D1 | N = 67 | N = 27 |  |
|  | 2.08 (0.11-9.9) | 1.87 (0.00-4.31) | 0.018 |
| D2 | N = 61 | N = 27 |  |
|  | 1.81 (0.08-6.55) | 1.67 (0.04-13.41) | 0.832 |
| D3 | N = 43 | N = 21 |  |
|  | 0.96 (023-4.54) | 0.74 (0.10-3.70) | 0.529 |
| D4 | N = 19 | N = 10 |  |
|  | 0.47 (0.16-1.2) | 0.71 (0.18-4.0) | 0.073 |
| D5 | N = 8 | N = 4 |  |
|  | 0.55 (0.20-0.75) | 0.72 (0.53-1.04) | 0.062 |
| ***ASCT*** |  |  |  |
| **Time from diagnosis to ASCT, months (median, range)** | 6.32 (3.57-38.21) | 6.02 (3.64-32.43) | 0.381 |
| **Time from mobilization to ASCT, days (median, range)** | 35.00 (12.0-183.0) | 30.00 (11.0-88.0) | 0.211 |
| **Infused CD34 cell, ×10^6^/kg (median)** | 3.38 (1.80-9.90) | 3.54 (2.00-11.00) | 0.938 |
| **Neutrophil engraftment** | 67 (100.0%) | 26 (96.3%) | 0.115 |
| Time to neutrophil engraftment, days (median, range) | 10 (1-21) | 10 (3-13) |  |
| **Platelet engraftment** | 64 (95.5%) | 25 (92.6%) | 0.348 |
| Time to platelet recovery, days (median, range) | 10 (1-32) | 10 (8-37) |  |
| **Relapse after ASCT** | 23 (34.3%) | 13 (48.1%) | 0.208 |
| **Median relapse free survival** | 18.91 (4.25-72.35) | 12.75 (6.57-52.67) | 0.220 |
| **Median overall survival (from ASCT)** | 30.04 (1.25-107.18) | 33.11 (0.50-85.07) | 0.514 |

Hb, hemoglobin; WBC, white blood cell; PLT, platelet; BM, bone marrow; ASCT, autologous stem cell transplantation; PB; peripheral blood; G-CSF, granulocyte colony stimulating factor

**Supplementary Figure 1.** Study design


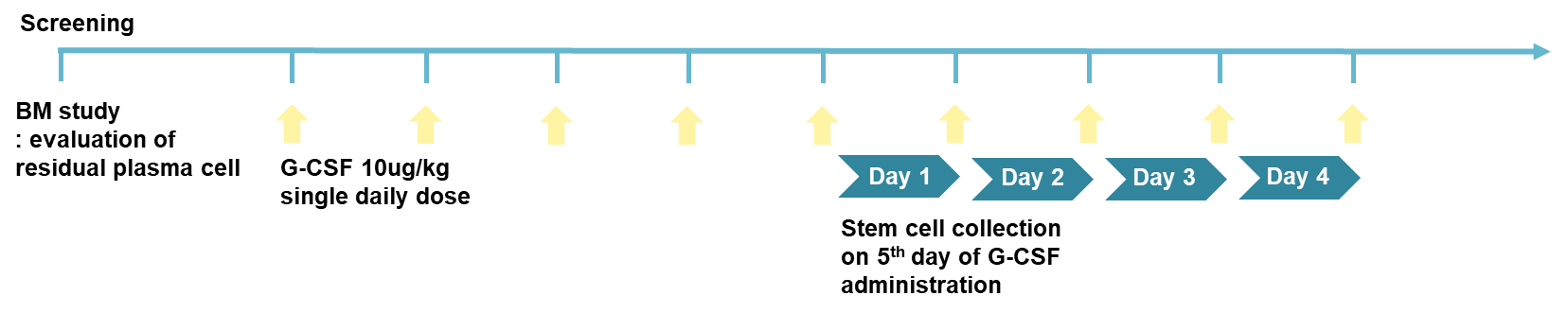


BM, bone marrow; G-CSF, granulocyte colony stimulating factor

**Supplementary Figure 2**. Consort diagram


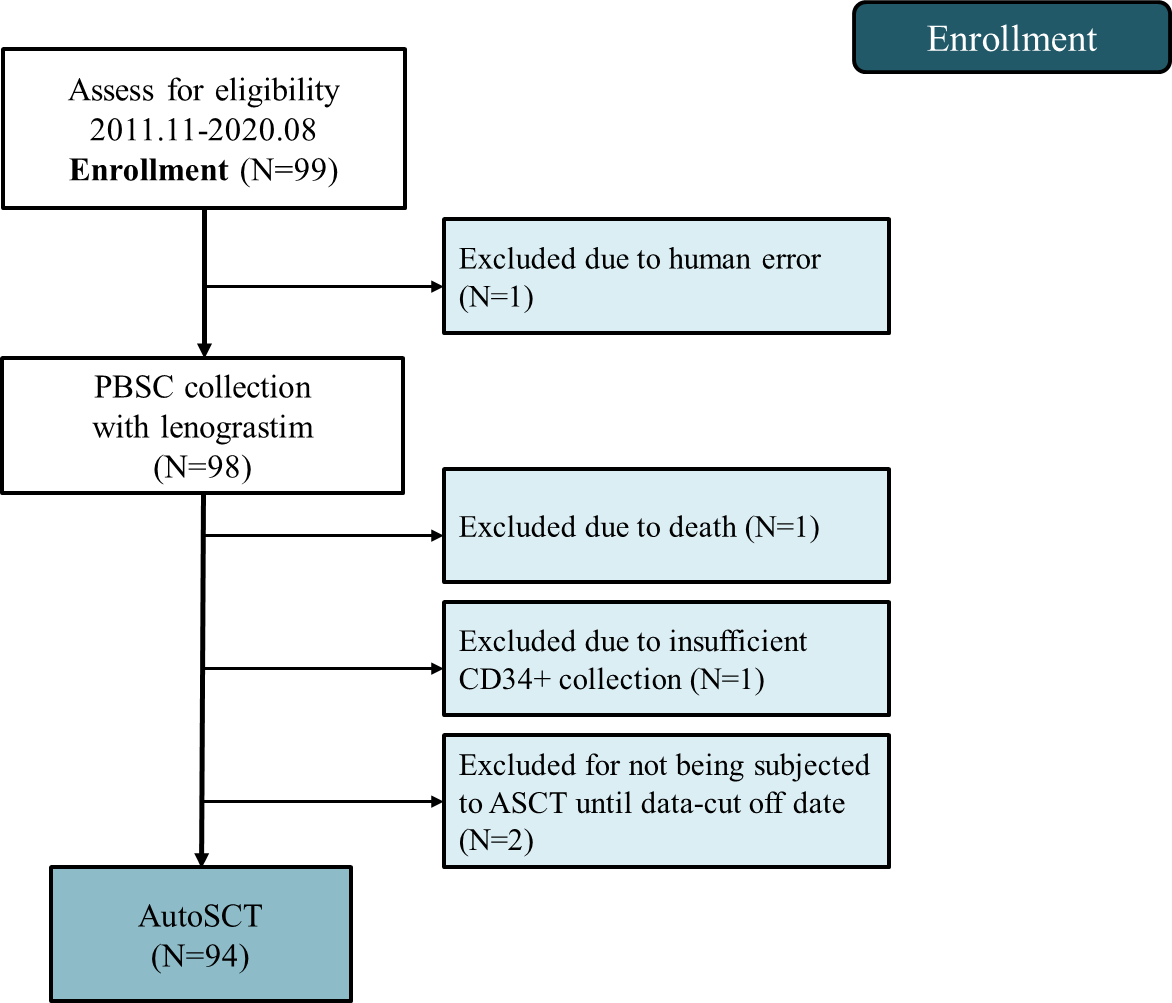


PBSC, peripheral blood stem cells; ASCT, autologous stem cell transplantation
